# Clinical placement experiences by undergraduate nursing students in selected teaching hospitals in Ghana

**DOI:** 10.1186/s12912-018-0325-8

**Published:** 2019-01-14

**Authors:** Confidence Alorse Atakro, Ernestina Armah, Awube Menlah, Isabella Garti, Stella Boatemaa Addo, Peter Adatara, George Sedinam Boni

**Affiliations:** 1 School of Nursing and Midwifery Faculty of Health and Applied Sciences, Christian Service University College, Post office box 3110, Kumasi, Ghana; 2 School of Nursing and Midwifery, Valley View University, Accra, Ghana; 3 School of Nursing and Midwifery, University of Health and Allied Sciences, PMB 31, Ho, Volta Region Ghana; 4 Volta Regional Hospital, PMB 374, Ho, Ghana

**Keywords:** Experiences, Undergraduate, Teaching hospitals, Critical thinking, Clinical Placement, Policy, Nursing students, Ghana

## Abstract

**Background:**

In meeting the global standard of patient safety, quality care and nursing leadership, countries are urged by the World Health Organisation to have a greater proportion of nurses educated to degree level or higher. However, some researchers have found that there are very little differences in competencies of diploma registered nurses and first degree nurses in some countries. University education in nursing remains problematic and there are many disparities in the programmes currently being offered in different parts of the world. Though teaching hospitals in Ghana are expected to assist in the training of undergraduate nursing students, there is limited scientific evidence on experiences of undergraduate nursing students in these teaching hospital environments. The purpose of this study was to explore the experiences of undergraduate nursing students in selected teaching hospitals in Ghana.

**Methods:**

A qualitative explorative descriptive design was used in conducting the study. Purposive sampling technique was utilised in collecting data from thirty-five undergraduate nursing students placed in two teaching hospitals in Ghana. Data were collected through a semi-structured interview guide and analysed manually by the research team. A thematic content analysis was used in analysing data.

**Results:**

Four main categories of themes were extracted from data. These themes were: 1. Feeling isolated in clinical placement. 2. Inadequate application of the nursing process. 3. Encounter with complex medical devices and complex conditions. 4. Inadequate application of physical examination by nurses.

**Conclusion:**

There were both positive and negative experiences by undergraduate nursing students in teaching hospitals in Ghana. The opportunity to see various clinical cases and also use complex medical devices were positive experiences for students. However, the undergraduate nursing students also experienced challenges of isolation in placement, inadequate application of the nursing process, and inadequate application of physical assessment by nurses. Undergraduate nursing students require varying levels of support, supervisory commitments and logistics provisions to learn skills such as physical examination and nursing process during placement.

**Electronic supplementary material:**

The online version of this article (10.1186/s12912-018-0325-8) contains supplementary material, which is available to authorized users.

## Background

The future of nursing lies in adequate preparations of nurses at first-degree level [1]. In meeting the global standard of patient safety, quality care and nursing leadership, countries are urged by the World Health Organisation (WHO) to have a greater proportion of nurses educated to degree level or higher [1]. Institute of Medicine (IOM) [2] recommended that 80% of Registered Nurses (RNs) should acquire and practice with a bachelor’s degree by the year 2020. A study by Aiken et al. [3] showed that surgical patients in Pennsylvania experienced lower mortality and failure-to-rescue rates in hospitals with higher proportions of nurses educated at the baccalaureate level or higher. In the study by Aiken et al. [3] nurses’ years of experience were not found to be a significant predictor of mortality or failure to rescue in surgical patients. Though undergraduate nursing students are being educated to have higher clinical competencies than their diploma counterparts, a study by Fero et al. [4] found that new graduate nurses had difficulties in meeting job expectations and struggled to make and implement independent nursing interventions. Research evidence available indicates that clinical placement is an important part of the undergraduate nursing programme in the preparation of students for entry into the nursing profession [5, 6] as it provides undergraduate nursing students with the opportunity to learn within clinical practice environments in real life situations [7]. Apart from the preparation of students for their professional roles, clinical placement affords undergraduate students opportunities to apply knowledge and skills they have learned in universities [8, 4]. A study by Baraz et al. [7] showed that undergraduate nursing students experience challenges that derail their training during clinical placements. The inability of undergraduate nursing students to demonstrate higher levels of clinical competencies may be a result of the myriads of challenges that confront them in clinical practice [9, 5–7]. A study in Iran found that undergraduate students did not acquire the right competencies during clinical placements as a result of inadequately prepared clinical teachers and poor application of theory in practice by practising nurses [7]. Undergraduate nursing students report similar difficulties in clinical learning environments across many countries [10]. Several studies have stated that the incompetence of instructors, negative attitudes of staff, inadequate students support from hospital management and shortages of positive role models [11, 12, 5] were predominant among the challenges experienced by undergraduate nursing students during clinical placements. Despite challenges experienced by undergraduate nursing students in placements, there are also positive experiences found by some investigators. A supportive relationship with some nursing staff helped students to internalise nursing roles expected of them [13]. The exposure of undergraduate nursing students to various clinical scenarios have also helped in developing their clinical competencies [14]. There is an increase in institutions offering first degree programmes in nursing due to the recommendations of the WHO to have higher proportions of degree nurses in practice [15]. However, university education in nursing remains problematic with many disparities in the programmes currently being offered in different parts of the world [1]. A study in Taiwan by Cheng et al. [16] found that undergraduate nursing students had textbook knowledge but lacked the clinical experience to apply the knowledge to practice. Many graduate nurses usually find themselves unfit in the clinical setting in the first year after completing university and consider changing jobs within the same period [17]. Though teaching hospitals in Ghana are expected to assist in the training of undergraduate nursing students, there is limited evidence on experiences of undergraduate nursing students in these teaching hospital environments. There is a need to conduct studies into undergraduate nursing education in Ghana in order to help produce nurse leaders who can have higher critical thinking skills and also participate in the development of policies for the health sector. The purpose of this study is to explore clinical placement experiences by undergraduate nursing students at selected teaching hospitals in Ghana.

## Methods

### Design

The study used a qualitative explorative descriptive design that allowed understanding of the experiences of undergraduate nursing students in selected teaching hospitals in Ghana. These teaching hospitals were Komfo Anokye Teaching Hospital (KATH) and Korle-Bu Teaching Hospital (KBTH). Qualitative research affords an understanding of a phenomenon and usually represents the participants’ world [18]. Hence undergraduate nursing students’ world within teaching hospitals was described.

### Setting

This study was conducted at the medical, surgical, paediatric and emergency units of Komfo Anokye and Korle-Bu teaching hospitals in Ghana. Korle-Bu Teaching Hospital (KBTH) is the largest teaching hospital in Ghana and located in the national capital Accra whilst Komfo Anokye Teaching Hospital (KATH) is the second largest teaching hospital located in the Ashanti region of Ghana. The aims of teaching hospitals in Ghana include provision of medical education, research and quality health services [19]. Teaching hospitals also serve as referral centres for regional hospitals in Ghana [19].

### Population and sampling technique

The population of this study was undergraduate nursing students placed in Komfo Anokye and Korle-Bu teaching hospitals. A total of 79 students were placed in the medical, surgical, paediatric and emergency units of KBTH and KATH at the time of this study. Thirty-five (35) participants including nineteen (19) females and sixteen (16) males aged between twenty-three (23) and thirty- two (32) years were involved in the study. Twenty-five (25) participants were in their fourth year and ten (10) participants were in their third year. Inclusion criteria were undergraduate nursing students who were in their third and fourth years and had at least three placement experiences within a teaching hospital. Third and fourth years provided richer experiences within the teaching hospital. Exclusion criteria were registered general nursing students who were not offering the first degree as a programme. Undergraduate students who were in their first or second years were also excluded. Purposive sampling technique was used to recruit undergraduate nursing students who met the inclusion criterion. A list of undergraduate nursing students placed in medical, surgical, pediatric and emergency wards was requested from unit nurse managers. Only students who met the inclusion criterion were selected to take part in the study. Data saturation was reached after interviewing 35 participants.

### Data collection and management

Data collection was done through semi-structured interviews. Data were collected from January to March 2017. Date, time and venue of interviews were at the convenience of respondents. Interviews lasted between 60 min and 90 min. The interviews focused on experiences by undergraduate nursing students placed in selected teaching hospitals in Ghana. Questions asked during interview sessions included the following: 1. Describe experiences of clinical supervision in wards of this hospital. 2. Describe your experiences with learning the nursing process in wards of this teaching hospital. 3. Describe the advantages of placement in this teaching hospital. 4. Describe your experience in learning physical examination in this hospital. Additional interview questions can be found in the attached file (Additional file 1). All interviews were conducted in English by authors. Probes were used to elicit detail understanding into experiences of undergraduate nursing students in teaching hospitals. A tape recorder was used in recording interviews. Verbatim transcription was done by authors. Transcripts were password protected to ensure confidentiality. All copies of data were saved on a pen drive purposely acquired for study purposes. Participants were identified with codes to ensure confidentiality and anonymity.

### Data analysis

Data analysis was conducted with thematic content analysis. Data were analysed manually by the research team. Holloway and Wheeler’s [20] data analysis pattern was used during data analysis. This pattern takes the following form; validating recorded data, transcribing data, cleaning and coding data. Codes were found during readings of transcripts. Similar codes were used to create families and similar families grouped together as themes [21].

### Rigour

Trustworthiness in this study was ensured by member checking and peer debriefing during data analysis. Member checks and peer briefings facilitated understandings into the participants’ experiences within the teaching hospital facility. Field notes and transcripts were also discussed among the research team to ensure correct interpretation of data.

### Ethical consideration

Institutional and unit permissions were obtained. Ethical approval for this study was granted by the Committee on Human Research Publication and Ethics (CHRPE) of Komfo Anokye and Korle-Bu Teaching hospitals. Permission was also granted by universities whose students were involved in this study. Individual informed consent was obtained from all undergraduate nursing students who participated in the study. Anonymity and confidentiality were explained to participants and they were assured that withdrawal will not in any way attract sanctions. Participants were given informed consent form to fill out and sign. Participants were identified with codes to maintain confidentiality and anonymity. Researchers made sure that this study did not cause any physical, psychological or emotional harm to any participant.

## Results

Thirty-five (35) participants including nineteen (19) females and sixteen (16) males aged between twenty-three (23) and thirty-two (32) years were involved in the study. Twenty-five (25) participants were in their fourth year and ten (10) were in their third year. Thirty-three (33) participants were single whilst two (2) were married. Twenty-seven (27) participants were Christians whilst eight (8) were Muslims.

Four thematic categories were extracted from data. These categories were 1. Feeling isolated in clinical placement. 2. Inadequate application of the nursing process. 3. Encounter with complex medical devices and complex conditions. 4. Inadequate application of physical examination by nurses.

### Feeling isolated in clinical placement

Many undergraduate nursing students (30 out of 35) interviewed indicated that they felt isolated during their placements in the teaching hospital. University supervisors did not visit clinical sites regularly and staff nurses did not also have time to teach students. This was evident in the following statements by a participant:

*Here we are not being monitored. I wish nurses will always be with us when we are trying our hands on procedures but it barely happens. The nurses are always busy with other things. You are mostly asked to perform tasks on your own. If you request for supervisions, they will call a diploma nurse to do it and it looks like we degree student don’t know anything. It is very embarrassing. To avoid embarrassment, you just try to do what you can, but sometimes you can feel isolated. It’s like you are on your own [P7].*

Another participant said:

*Some staff nurses actually tell us that they are not paid to teach us. When you ask questions they will tell you to google it but when they see you using the phone to look for the information, they will call you WhatsApp nurses. Our school supervisors don’t usually come here but when they do, nurses tell them that we are always on our phones. It is disturbing. We don’t know what to do to make them happy so we just try to learn on our own [P11].*

Yet another students stated:

*I think the nurses in the hospital are not paid for their services and the selected supervisor cannot be in all wards at once. So supervision is not strict. Supervisors in the hospital rarely come to my ward and staff nurses don’t mind us most of the time. Sometimes I feel we are just on our own [P35].*

P6 said:

*There are more duties for staff nurses so staff nurses don’t have time for students. They are always busy with other things. You have to learn things on your own. Sometimes when you follow some nurses during procedures, you will be lucky to be taught one or two things. Nurses always want to finish their work before the next shift nurses report to work. They will prefer to send us on errands the whole day to make them accomplish their work. When we are sent on errands we also do other things. Most of the times we don’t really learn much at the end of the day. It is like everybody for himself and God for us all.*

Many students (31 out of 35) indicated that there were negative attitudes towards them in the teaching hospital and this made them feel isolated. Majority of nurses working in the wards were diploma nurses who were not interested in teaching degree student nurses. Undergraduate student nurses were also branded as theory nurses which usually embarrassed them. A participant said:

*When the staff nurses who completed nursing training see you in white, they start showing bad attitude towards you. Some of them say that in a few years we will be seniors over them. So sometimes it looks like they are envious so they don’t want to teach us. I get worried when this happen. It means they are not really ready to teach us. All we want is to learn but it does not happen most of the time. If you are not able to do something, they will just call a diploma student nurse to do it. I think they don’t like us [P15].*

Another participant said:

*The staff nurses don’t like us. Especially the females. They tell us that we don’t know anything and don’t respect. Some of them call us theory and WhatsApp student nurses. They sometimes use phones whilst at work but if we also do it then they call us WhatsApp nurses and say we are not serious. It worries me when they do that because it is not all of us who do the WhatsApping [P31].*

Yet another participant said:

*It seems the white dress infuriates them. When they see us in the white dresses, they usually don’t want to look at our faces. They call us theory nurses. They always say the diploma students know more than us. It is as if we have done something wrong by doing degree. It disturbs me psychologically so most times I don’t want to mix with people on the ward [P11].*

### Inadequate application of the nursing process

Majority of students (32 out of 35) stated that there was a poor application of the nursing process in the wards. Many students mentioned that they did not have the logistics for care plans. Nurses were not also using care plans to care for clients. Therefore components of the care plan such as nursing diagnosis remained abstract to most students. An undergraduate nursing student expressed this in the following statements:

*We don’t apply the care plan that we learn in the classroom. Even the forms are not available in the wards. I wish we could apply the care plan every day. It would have helped us very much even in our practical exams. The care plan looks mostly abstract to me. I find it difficult to come up with nursing diagnosis automatically [P34].*

Another participant indicated:

*I think the nursing process is for exams. When they are teaching us, they tell us that we should apply when caring for our clients. But here no one applies the care plan. It is only when we are writing exams that we apply. May be the workload is too much to apply the care plan [P24].*

Another first degree nursing student said:

*We are taught care plan in school but since I have been coming here, I have not seen any nurse using the care plan. It is difficult to learn the care plan well because it is not applied during practice. I wish it could be applied by nurses so that we can also benefit from it during clinicals [P18].*

Another student said:

*Sometimes I wonder whether we are just learning for exams. It’s like we are learning just for examination. Nurses do not pass through all stages of the nursing process to care for patients here. It was when I was having practical exams that I saw there was a form for the nursing process. Even the nursing diagnosis is difficult to come by. We usually memorise a number of nursing diagnoses when it’s time for exam. So whether the patient has it or not that’s what we apply. This happens because it is not practicalised for us during clinical practice [P24].*

A participant said:

*In fact no one talks about the care plan when we are working. We hear of care plans when we are coming for practical exams. We don’t even see the forms here. The forms are brought by our school when we have practical exams. When we finish with practical exams, the lecturers will send the rest of the care plan forms back to school to wait for the next practical examination* [3]*.*

### Encounter with complex medical devices and complex conditions

Majority of respondents (31 out of 35) stated that they had the opportunity to see complex medical devices and complex conditions in the teaching hospitals. Undergraduate nursing students indicated that they did not see very complex medical devices or conditions in district hospitals. It was, therefore, an opportunity to find these conditions and devices in the teaching hospitals where they were placed. A participant stated:

*As compared to district hospitals, we see complex devices and sicknesses here. Some of the conditions such as tracheostomy we see it only here. They are not available in district hospitals. The first time I saw a patient with a colostomy bag was here. These are the advantages of being placed in a teaching hospital* [22]*.*

Another students said:

*It is only here that we see all the big and serious diseases that are taught in the classroom. You will not find these in smaller hospitals. It is a good experience to see some of these conditions live. When you are writing an exam and it comes you can try and remember somethings [P29].*

Another student stated:

*You know there are so many conditions here. Unlike the district hospital, you cannot find these conditions there. When we go back to school and other students are talking about things they have seen during the clinicals, we can also say what we have seen* [23]*.*

One undergraduate student stated:

*It is very difficult to see some of these cases in the district hospitals because they are usually referred here. So when we are placed here we get the opportunity to see them live and care for them. I think every students must experience the teaching hospital because of the conditions and machines [P30].*

### Inadequate application of physical assessment by nurses

Many students (29 out of 35) interviewed indicated that they did not apply physical assessment lessons learnt in the classroom. Students stated that all they did was checking of vital signs. They were not usually taught other aspects of physical assessment such as percussion and auscultation during clinical placements.

One students said:

*As for physical examination, we don’t do it into detail. It is only vital signs we do as students here. We mostly wait for the vital signs time to do vital signs. We don’t really do all those percussion and auscultation that we were taught in the classroom [P2].*

Another student said:

*We only use the stethoscope to check the blood pressure. We don’t use it to do any other thing. Auscultation is still abstract to me. Even the practicing nurses themselves don’t do the percussions and auscultation. It looks like it is for doctors [P20]*

P24 said:

*It is mostly doctors I see using the stethoscope to auscultate. I think the nurses don’t have time for that or may be they don’t know that aspect very well. I wish I knew how to apply auscultation and percussion learnt in classroom [P6].*

Another student indicated:

*I even feel shy to try auscultating a patient because the other nurses may ask me whether I am a doctor. Meanwhile we are taught to do all these for our patients. But we don’t see it being done by nurses for patients in the wards [P33].*

Another participant said:

*Hmmmmm. As for that auscultation and percussion we don’t do it. Who will even teach you that one here? We don’t even see any nurse doing it so we can’t ask them to teach us [P34]*

## Discussion

Discussion of results in this study is done in line with themes extracted from data.

### Feeling isolated in clinical placement

Evidence in this study showed that undergraduate students felt isolated during clinical placements in the teaching hospital environment. This is consistent with a study in Malawi into challenges of undergraduate students in placements in which researchers identified a theme of ‘lost sheep’ among students [22]. Some reasons attributed to this phenomenon in literature are; absence of preceptors, inadequately prepared preceptors and inadequate supervisions [5, 23]. The feeling of isolation may also be partially due to the intergenerational gap between preceptors and undergraduate students [24]. Many staff nurses referred to undergraduate nursing students as WhatsApp nurses because they were always seen browsing with their phones. A study by Foley et al. [24] found that majority of today’s university and college students belong to the Millennial Generation (also called Generation Y), while most preceptors are either Baby Boomers or Generation Xers. Values and expectations within the nursing discipline are deeply rooted in the traditions of nursing practice and as the younger generation brings new ideas to the practice setting, clashes between the various generations occur [24]. These generational differences must be acknowledged by all stakeholders including students, lecturers, clinical supervisors and preceptors. Students, lecturers, clinical supervisors and preceptors should all be willing and ready to accommodate one another and find ways of relating to each other despite the intergenerational gap. Though there may be generational differences, there are certain commonly shared values and goals between undergraduate nursing students, supervisors and staff nurses. Students and staff nurses must be informed about possible generational differences in classrooms before clinical placements. Awareness of students own generational characteristics and that of their supervisors is necessary for adaptations within the clinical environment. A qualitative study by Baraz et al. [7] in Iran identified lack of teaching and learning support, lack of opportunities for learning, poor theory-practice integration, and poor interpersonal relationships between undergraduate nursing students, lecturers and ward staff as challenges undergraduate nursing students experienced in clinical placements. Similarly, a study into the world of undergraduate nursing students in Malawi by Msiaka et al. [22] found that undergraduate students were usually left unsupervised during clinical placements and lecturers only visited clinical settings occasionally. Majority of nurses in hospitals in developing and lower-middle-income countries are diploma and certificate registered general nurses who usually feel they did not have the required training to teach undergraduate student nurses. Promoting a positive practice environment for undergraduate nursing students is a shared responsibility of nurse educators, preceptors, nurse manages and staff nurses. The attitude of undergraduate students may determine the level of supervision and teaching they receive from staff nurses who may mostly be diploma registered general nurses. Students in this study indicated that ward sisters were more likely to teach them when they got involved in nursing care. It is important to be a participant rather than a spectator in the clinical setting. Undergraduate nursing students must strive to get the confidence and attention of supervisors by getting involved in the direct nursing care and be persistent in asking questions. In a study by Helgesen et al. [25], students pointed out that information failure and a lack of communication of objectives were reasons for underachieving in the clinical settings. Clinical nursing education in Ghana is currently facing challenges of poor coordination between hospitals and universities [23]. Challenges of clinical nursing education in Ghana include inadequate faculty supervisions and inadequate preceptor preparations [23]. Findings confirm the importance of student support during clinical placements. In the absence of adequate supervision, students may be engaged in trial and error and this has serious implications for patient safety. Several researchers have reported positive outcomes of collaborative preceptorship models between schools of nursing and health service where undergraduate nursing students increased confidence levels and were able to integrate skills with real-life situations [5, 23, 26]. Clinical education of undergraduate nursing students can become effective through collaboration between nursing schools and health service, where staff nurses and faculty members regularly participate in a collaborative preceptorship or clinical teaching partnerships. Undergraduate nursing students will hardly feel isolated during placements if they have adequate supervisions by lecturers and preceptors.

### Inadequate application of the nursing process

Though the nursing process has become the gold standard of nursing care in hospitals and in communities [27], many undergraduate student nurses interviewed indicated that there was an inadequate application of the nursing process in teaching hospitals where they were placed. There seems to be a gap in the application of the nursing process in many hospitals in Ghana including teaching hospitals. Implementation of the nursing process in many low and middle-income countries has not been very effective [27]. It is observed that some aspects of the nursing process appear to be poorly implemented, particularly, the aspect of nursing diagnoses [28, 29]. The nursing diagnosis stage is the basis for nursing interventions [28], therefore errors in diagnoses could lead to inappropriate interventions that would fail to address patient’s health problems [27]. Though teaching hospitals were established and positioned to provide the best clinical education to health practitioners of all categories including nurses, student nurses in this study did not see nurses using the nursing process during their placements. They also realised that supplies such as care plan forms to implement the nursing process effectively were absent. Apart from inadequate knowledge of aspects of the nursing process, there could be other contextual factors that pose challenges to the effective implementation of the nursing process in Ghanaian teaching hospitals. In Ghana, the nursing process was introduced into the nursing curriculum in the 1980s, but its implementation in practice has been below expectation [27]. Some challenges that confront the nursing process implementation in Ghana include inadequate staffing, inadequate financing, time constraints and knowledge gap [27]. Afolayan et al. [28] noted that despite good theoretical knowledge following training, nurses in a Nigerian hospital did not implement the nursing process and blamed it on inadequate staff, work overload, and management’s inability to provide the needed materials. However, a case study in Kenya showed that following a training on the nursing process, a tremendous improvement in the application of the nursing process and documentation in hospitals were recorded [30]. Improvements in the application of the nursing process could be sustained with further interventions. It has been proposed that a sustained improvement in the use of the nursing process is achievable if nurse managers put in strategies that lead to a mutual interconnection of people, environment, process and technologies [29]. The nursing process could be simplified to meet cultural contexts for implementation in Ghana as done in Kenya. Continuous in-service training for nursing staff of teaching hospitals is necessary to make them utilise the nursing process in caring for clients. Supervisory commitment and logistic provisions are also necessary to sustain implementation of the nursing process [28, 30]. Practising nurses can only teach students if they continuously utilise all aspects of the nursing process under constant supervision and support from nurse managers. There is need for the Ministry of Health in Ghana and its service delivery agencies including the Ghana Health Service, teaching hospitals and the faith-based health facilities to partner academic institutions to develop a national framework for the use of the nursing process, and develop a support mechanism for a sector-wide use of this important and core nursing tool to enhance quality nursing care delivery [27].

### Encounter with complex medical devices and complex conditions

Majority of the undergraduate nursing students interviewed indicated they had the opportunity to see complex disease condition and complex medical devices as a result of placements in teaching hospitals. Extensive clinical exposure of undergraduate students is crucial for their clinical experience. A study by Alhaqwi et al. [14] concluded that exposure of students to a variety of clinical experiences usually occurs in teaching hospitals and contributes positively to the development of their clinical competence. There are usually sufficient clinical cases that students can learn from during placements in teaching hospitals. Students’ clinical learning may be enhanced when they are exposed to a variety of clinical cases that are new to them [14]. However, the availability of clinical cases and modern equipment or devices alone do not ensure clinical learning. Large facilities with rich learning opportunities must also provide a conducive environment for students learning. Evidence available shows that a rich clinical environment with less supervision will not lead to improvements in students competencies [5, 23]. Establishing caring relationships with students is key to creating caring learning environments [5]. Early exposure of students to a variety of clinical cases helps them to develop a positive attitude towards patient care [14] but must not be done without the necessary clinical support system.

### Inadequate application of physical examination by nurses

Participants in this study stated that they were not taught effectively regarding physical examination during their placement in teaching hospitals. This is consistent with a study by Duvivier et al. [31] who found that factors affecting the learning of physical examination by students negatively were lack of supervision, uncertainty about tasks and expectations, and unfavourable clinical learning environment. Practising physical examination in the clinical setting is crucial to students learning of health assessment. Proactivity and participation are driving forces for learning physical examination in the clinical site [31]. Merely placing students in a clinical setting does not automatically lead to learning and offering learning opportunities do not mean that students will automatically make use of them in improving their clinical competence regarding physical examination [5, 23, 31]. Continuous educational programmes in physical examination could be organised for practising nurses. These education programmes will improve their competence and confidence in the practice of physical examinations. Workshops will also help disabuse the minds of practising nurses that some aspects of physical examination such as percussion and auscultation are sole responsibilities of medical officers. Daily ward conferences where nurses, medical officers and student nurses discuss clinical cases could be organised by ward managers. These ward conferences will help practising nurses and students improve their skills such as auscultation and percussions in various disease conditions. Students themselves must show interest in learning physical assessment even if it means asking house officers and medical officers for help in that regard. A study by Duvivier et al. [31] found that undergraduate clinical training in itself was inadequate in terms of skills taught and competencies achieved. Supervisors and preceptors can contribute enormously in setting clear objectives for undergraduate nursing students for learning physical examination in the clinical settings. It must, however, be stated that supervisors cannot create good clinical learning environment without input from learners and no student no matter how proactive can force learning to happen without the prerequisite support from the supervisor [31]. Lecturers who teach physical assessment could find time to participate in ward rounds in order to keep their own competencies updated. Ward managers could provide teaching and learning opportunities for nursing lecturers on the wards without interferences. This concept of interdependence described by Billett [32] where individual agencies and social aspects of learning environments come together to make the learning of physical examination by undergraduate students effective is an important concept that should be utilised to the benefits of students.

## Conclusion

Undergraduate nursing students had various experiences during their clinical placements in teaching hospitals. There were both positive and negative experiences by undergraduate nursing students in teaching hospitals in Ghana. The opportunity to see various clinical cases and also use complex medical devices were positive experiences for students. However, a number of challenges were also experienced by undergraduate nursing students. These challenges included a feeling of isolation in clinical placement, inadequate application of the nursing process, and inadequate application of physical assessment by nurses. Undergraduate nursing students require varying levels of support, supervisory commitments and logistics provisions to learn skills such as physical examination and nursing process during placement.

## Recommendations

The following recommendations are made to help improve the clinical education of undergraduate nursing students in Ghanaian teaching hospitals:

1. Though undergraduate nursing students are exposed to a variety of complex conditions and medical devices in teaching hospitals, they require varying levels of support, supervisory commitments and logistics provisions to learn skills such as physical examination and nursing process. These support systems will make students feel less isolated during placements in teaching hospitals.
2. A national framework for the practice of the nursing process where it is modified and simplified to suit the cultural context of Ghana can help improve implementation of the nursing process. This framework must, however, be supported by continuous training, supervisory commitments and logistics provisions.
3. Results of this study could be used by stakeholders to improve undergraduate nursing education in Ghana by formulating policies on clinical studies in teaching hospitals taking into consideration the experiences of the students.
4. Daily ward clinical case conferences can be scheduled where nurses, medical officers and students discuss and share ideas on assessment of patients with various diseases within the teaching hospital environment. Every member of the healthcare team in each ward including nurses must be assisted to be competent in the physical assessment of clients because effective patient management may not be possible without it.
5. Adequate information on intergeneration differences must be provided to students and clinical staff before students’ placements within teaching hospitals. Awareness of differences is necessary for effective adaptations with the clinical environment for the benefits of students. Clinical staff and students should also be made aware that despite the intergenerational differences, there are several commonly shared values and goals between them.
6. Universities and teaching hospitals should collaborate and institute regular continuous education for staff nurses on collaborative preceptorship or clinical teaching partnerships. This will bring about good working relations between universities and health care agencies.

## Additional file

Additional file 1: Supplementary file 1 - Interview guide. (DOCX 16 kb)

## Abbreviations

CHRPE: Committee on Human Research Publication and Ethics
IOM: Institute of Medicine
KATH: Komfo Anokye Teaching Hospital
KBTH: Korle-Bu Teaching Hospital
RN: Registered Nurses
WHO: World Health Organisation
