# Supplementary material for: Clinical placement experiences by undergraduate nursing students in selected teaching hospitals in Ghana
Source: BMC Nurs. 2019 Jan 14;18:1. doi: 10.1186/s12912-018-0325-8 (PMC6332612; doi:10.1186/s12912-018-0325-8)
Supplement: Supplementary file 1 — Interview guide. (DOCX 16 kb) [file 12912_2018_325_MOESM1_ESM.docx]

**INTERVIEW GUIDE**

This study is being conducted to find out clinical placement experiences by undergraduate nursing students in selected teaching hospitals in Ghana. This interview guide contains open ended questions. You are expected to kindly provide genuine answers to the questions. The information you provide is confidential and will be used only for the purposes of this study. If you have any question, do not hesitate to ask the researchers. Your cooperation and participation until the completion of the interview is very necessary for the successful completion of the study. However your participation in this study is entirely up to you. You will not be penalized in anyway by refusing to participate. You will need to sign the attached consent form before we start the interview if you agree to be part of this study.

SECTION A: socio-demographic data

Kindly tick as appropriate:

1. Age of respondent.

a. 18 – 22years

b. 23 – 27years

c. 28 – 32years

d. 33– 37years

e. 38– 42years

f. 43-47 years

g. 48-52 years

h. 53-57 years

i. 58-62 years

j. 63-67 years

k. 68-72 years

l. 73-77 years

j. Other…………..

2. Gender

a. Male

b. Female

3. Religion

a. Islam

b. Christianity

c. Traditional Religion

d. Any other religion……………..

4. Marital Status

a. Single

b. Co-habitation

c. Married

d. Divorced

e. Widowed

f. Separated

g. Any other……………

5. Number of Children

a. None

b. One

c. Two

d. Three

e. Four

f. other, state…………..

6. Place of clinical experience

a. Korle-bu Teaching Hospital

b. Komfo Anokye Teaching Hospital

7. Indicate the name of your university………………………………………………….

8. Indicate your level or year.

a. level 100/first year

b. level 200/second year

c. level 300/third year

d. level 400/fourth year

9. Indicate your unit of clinical experience…………………………………………………..

INTERVIEW SECTION

1. Describe experiences of clinical supervision in wards of this hospital.
2. Describe your experiences with learning the nursing process in wards of this teaching hospital.
3. Describe the advantages of placement in this teaching hospital.
4. Kindly tell be about any disadvantages of being placed in a teaching hospital.
5. Describe your experience in learning physical examination in this hospital.
6. Explain what you will like changed positively for your clinical experience in a teaching hospital.
7. Describe any clinical experiences that I have not asked about that you feel is important to note in this study.
